# The Diagnosis and Prognosis of Acute Pneumonia—I

**Published:** 1910-08-20

**Authors:** J. Walter Carr

**Affiliations:** Physician to the Royal Free Hospital.


### Hospital Clinics.

### THE DIAGNOSIS AND PROGNOSIS OF ACUTE PNEUMONIA—I.

By J. WALTER CARR, M.D., F.R.C.P., F.R.C.S., Physician to the Royal Free Hospital.

A Lecture delivered at the Polyclinic, Chenies Street, W.C., on Thursday, July 14, 1910,

Gentlemen,—You will forgive me for dealing to-day with one of the most familiar and common diseases, but, after all, I think it is these everyday diseases to which we need to pay special attention. It is a mistake always to be referring to the rarer conditions, which, however interesting they may be, we may not encounter for years. So perhaps I need hardly any excuse for speaking of the diagnosis and prognosis of acute pneumonia.

It may be truly said that there are few, if any, diseases which present such marked and unmistakable characteristics as does pneumonia in the majority of cases—the abrupt onset, the sharply-defined symptoms, the well-marked physical signs, the tempestuous course, the sudden crisis, the remarkable change, hardly paralleled by any other disease with which we are familiar, except perhaps typhus fever, fortunately now practically extinct in this country, by which in the course of a few hours the patient, after being apparently desperately ill, is on the high road to convalescence. I say there is no other disease which presents such unmistakable features, and which from the earliest history of medicine has attained more definite recognition. Yet in this very fact there lies a danger; a danger that because, in the majority of instances, the disease is so remarkably well marked and distinctive, we may miss the atypical and irregular cases. For, after all, such cases, although no doubt they are a minority, yet constitute a very considerable and important minority; and it has been my lot to see, at different times, a considerable number of examples of acute pneumonia in which, unfortunately, an erroneous diagnosis has been made. The usual mistake is to miss the disease when present rather than to diagnose it when it is not present.

### Overlooking Pneumonia.

Let us consider how the mistakes arise. We will first deal with symptoms, or rather with the comparative absence, in many cases, of symptoms. It is notorious that in old people with so-called secondary pneumonia, *i.e.* the pneumonia which so frequently terminates life in cases of cancer, diabetes, and, most of all, Bright's disease, the symptoms are often extremely slight, and those which are present may be highly misleading. Let me describe a by no means imaginary case: a man seventy years of age, who, it may be, for two or three years past has been suffering from slight signs of contracted granular kidney. He has increased frequency of micturition, his urine is of low specific gravity and contains a trace of albumen. For some time past he has been getting rather thinner and a little weaker; still he has had fairly good health until one morning, when he says he has not slept very well, that he has no appetite for his breakfast, and that he will stay in bed. He thinks he got a slight chill when he was out the previous day. You see him towards evening, his tongue is furred, he has complete anorexia, and perhaps a little cough, but this is so slight that it is easy to ignore it altogether. He does not complain of pain. His temperature is only 100°; yet how significant is even such a comparatively slight rise of temperature in an old man, and especially in one with signs of renal disease. The pulse is about 90, and the respirations are accelerated to 30 per minute, but the breathing is so quiet that unless you make a point of counting the number of respirations you will hardly notice that there is appreciable dyspnœa. You see him again the following morning; he has had a restless night, his mind has been wandering, and now he is obviously passing into low delirium; he mutters, picks at the bed-clothes, does not recognise you, and asks who you are; he is uncertain as to where he is. He absolutely refuses all food. His tongue is no longer merely furred, it is dry. His temperature has risen to 100°·5, the pulse is 100, respirations 35. Twenty-four hours later you see him again; he is obviously rapidly sinking, and is unconscious, the extremities are cold, the temperature has fallen to 99°, and the pulse is 110 and very irregular, the respirations are 40 and extremely shallow. A few hours later he dies. Throughout the whole short course of the illness he has never complained of pain, there has been very little cough, probably no expectoration—though there may be a little prune-juice sputum—and there has been no obvious dyspnœa. Examination is difficult, he resents it and it exhausts him; you cannot get him to sit up or to breathe deeply, and even if you can make a satisfactory examination the usual signs are by no means marked. There is perhaps some impairment of resonance at one base with rather weak breath sounds and some crackles on inspiration, that is all. During the last twenty-four hours of life the man is too ill to be examined, and no doubt many an old person passes away in this manner, and no one ever quite knows the exact cause of death. Yet if a post-mortem be made you will find the greater part of the lung in a state of complete red, or even grey, hepatisation. This is one class of case, and examples of it are numerous.

### Misleading Symptoms.

Let me pass on to some other classes in which the symptoms are not so much absent as misleading. First of all, the cases in which, predominantly at any rate, the symptoms are abdominal. Again let me outline the chief features. The patient, either an adult or a child, is taken suddenly ill with severe abdominal pain; at the same time there is vomiting, for a day or two perhaps almost incessant; there is much abdominal rigidity, also well-marked constipation. You find considerable rise of temperature, but this you naturally associate with the probability of some acute peritoneal inflammation—*e.g.* acute appendicitis, or acute inflammation in connection with the gall-bladder. Such a patient is perhaps lucky at the present day if he escapes with an unopened abdomen. There are perhaps comparatively few extra-abdominal diseases for which, at some time or another, the abdomen has not been opened. I have known it to be opened for mitral obstruction, for infective endocarditis, for the gastric crises of tabes, and for many another condition. For acute pneumonia the abdomen has been opened, and no doubt will be again. How can we guard against this disastrous occurrence? Solely, I think, by remembering the danger. We rarely make a mistake when we are aware that a mistake is likely to be made. Mistakes are due either to ignorance or to thoughtlessness. When we are confronted with a case of what appears to be an "acute abdomen," if we remember that not only may it be one of a dozen different abdominal conditions, but it may also conceivably be the earliest stage of acute lung disease, then I think this disastrous mistake of opening the abdomen for acute pneumonia will comparatively seldom, if ever, be made. If on inquiry we find that pain is definitely on one side, if we notice that the respiration rate is markedly increased, if on observing the patient we find there is a little cough which obviously increases the pain, we begin to think of the possibility of pneumonia. When once we think of it, we shall probably diagnose it, if it is present.

### The Cerebral Type.

The second type of case is that in which the cerebral symptoms predominate, and these occur especially in children. Again let us get a mental picture of such a case. The child is taken suddenly ill, perhaps with a convulsion; then follow vomiting, continuing for a day or two with great frequency, headache, possibly delirium (note, however, that the two do not co-exist); the patient whilst delirious does not complain of his headache as he does in a case of meningitis. Possibly even, to make the case more misleading, there is a squint. The child may have had the squint since birth, or it may be associated with the acute illness. Head retraction is sometimes present, and very often the patient is apparently more or less stuporous. What more do we want to complete the picture of acute meningitis? The resemblance is increased by the fact that, for the first day or two at any rate, cough is slight or altogether absent; very likely there is no complaint of pain in the chest, and on physical examination even for several days there may not be the slightest indication of pulmonary consolidation. Fortunately we do not open the skull for acute meningitis as a rule, and therefore the child escapes this danger; at the worst probably he will only have to endure an unnecessary lumbar puncture.

How are we to avoid in these cases the risk of an erroneous diagnosis? Remember that when a child is taken suddenly ill in this fashion, without any warning or apparent reason, it is more likely to be pneumonia than it is to be acute meningitis. There are not many cases of meningitis which begin so acutely as this. Notice the respiration rate. In a child it is almost certain to be markedly increased. The breathing may be very quiet—so quiet that you do not notice its increased rapidity unless you set out to count it. In all probability the alæ nasi will be working as well. There may be, although this is less frequent, a herpetic eruption on the lips. But it is the increased rate of the breathing and the working of the alæ nasi which should excite suspicion; and, as before, if one's suspicion is aroused that a mistake is possible, one is not likely to make it.

### Some Further Points.

I will refer to one or two other misleading conditions. Remember that it is very easy for delirium tremens to mask an acute pneumonia, and that it is not unusual for pneumonia, like any other acute disease, to give rise to delirium tremens. In these cases examination is quite impossible; the patient will not allow it. The sputum is often unobtainable. Always, however, note the temperature; it may not be easy to take, but you must get it. Remember that uncomplicated delirium tremens does not cause fever, so that if the patient has a high temperature there is something more to be thought of. I may say, also, that pyrexia is of the utmost significance in all maniacal or delirious conditions. Unfortunately there have been cases ere now in which patients have been sent off to a lunatic asylum because they were supposed to be suffering from delirium tremens or acute mania, whereas the mental condition was really due to an acute febrile disorder, often pneumonia. Such a mistake is, of course, an exceedingly grave one, and ought always to be avoided; it would be if more regard were paid to the temperature.

In passing I may note that typhoid fever in its earlier stages may present very much the clinical aspect of pneumonia. I believe this applies more to typhoid fever as met with abroad than in this country. Still I have seen cases in which the mistake has been made; not a very serious one perhaps unless, as I have known to happen, the medical man has given a strong aperient and has assured the friends he would not have done so if he had thought the case was one of typhoid fever, and yet subsequent events have shown that he was wrong. Of course, the prestige of that medical man among the friends and relatives naturally fell. The development of events after the first week or two will show that we are dealing with something besides pneumonia, although the latter may also be present.

Here, too, I may note a point about the expectoration. There is no symptom which is more significant and often helpful in diagnosis than the rusty sputum of acute pneumonia. When it is present never ignore it. But remember that in a considerable proportion of the cases the sputum is not characteristic. There may be some typical expectoration, but it is masked by that of associated bronchitis or pulmonary œdema. Do not, therefore, attach too much importance to the absence of rusty sputum.

(*To be continued.*)

